# Lightweight Anomaly Detection Scheme Using Incremental Principal Component Analysis and Support Vector Machine

**DOI:** 10.3390/s21238017

**Published:** 2021-11-30

**Authors:** Nurfazrina M. Zamry, Anazida Zainal, Murad A. Rassam, Eman H. Alkhammash, Fuad A. Ghaleb, Faisal Saeed

**Affiliations:** 1 School of Computing, Faculty of Engineering, Universiti Teknologi Malaysia, Iskandar Puteri 81310, Malaysia; nurfazrina2@graduate.utm.my (N.M.Z.); anazida@utm.my (A.Z.); 2 Department of Information Technology, College of Computer, Qassim University, Buraydah 51452, Saudi Arabia; m.qasem@qu.edu.sa; 3 Faculty of Engineering and Information Technology, Taiz University, Taiz 6803, Yemen; 4 Department of Computer Science, College of Computers and Information Technology, Taif University, P.O. Box 11099, Taif 21944, Saudi Arabia; eman.kms@tu.edu.sa; 5 College of Computer Science and Engineering, Taibah University, Medina 42353, Saudi Arabia; alsamet.faisal@gmail.com; 6 School of Computing and Digital Technology, Birmingham City University, Birmingham B4 7XG, UK

**Keywords:** anomaly detection, one-class support vector machine, principal component analysis, wireless sensors networks, sensor data analysis

## Abstract

Wireless Sensors Networks have been the focus of significant attention from research and development due to their applications of collecting data from various fields such as smart cities, power grids, transportation systems, medical sectors, military, and rural areas. Accurate and reliable measurements for insightful data analysis and decision-making are the ultimate goals of sensor networks for critical domains. However, the raw data collected by WSNs usually are not reliable and inaccurate due to the imperfect nature of WSNs. Identifying misbehaviours or anomalies in the network is important for providing reliable and secure functioning of the network. However, due to resource constraints, a lightweight detection scheme is a major design challenge in sensor networks. This paper aims at designing and developing a lightweight anomaly detection scheme to improve efficiency in terms of reducing the computational complexity and communication and improving memory utilization overhead while maintaining high accuracy. To achieve this aim, one-class learning and dimension reduction concepts were used in the design. The One-Class Support Vector Machine (OCSVM) with hyper-ellipsoid variance was used for anomaly detection due to its advantage in classifying unlabelled and multivariate data. Various One-Class Support Vector Machine formulations have been investigated and Centred-Ellipsoid has been adopted in this study due to its effectiveness. Centred-Ellipsoid is the most effective kernel among studies formulations. To decrease the computational complexity and improve memory utilization, the dimensions of the data were reduced using the Candid Covariance-Free Incremental Principal Component Analysis (CCIPCA) algorithm. Extensive experiments were conducted to evaluate the proposed lightweight anomaly detection scheme. Results in terms of detection accuracy, memory utilization, computational complexity, and communication overhead show that the proposed scheme is effective and efficient compared few existing schemes evaluated. The proposed anomaly detection scheme achieved the accuracy higher than 98%, with O(*nd*) memory utilization and no communication overhead.

## 1. Introduction

With the advancement of digital technology from the past few decades, every digital equipment and appliance is expected to be embedded with tiny yet powerful device called sensor nodes. Furthermore, the wireless communication between physical items and sensors to exchange information for smart living in the future has been coined as the Internet of Things (IoT). In the world of modern wireless telecommunications, IoT is a revolutionary paradigm that is rapidly growing [1]. When these sensor nodes communicate together to collect a large amount of data from the targeted area via the wireless channel, they are called Wireless Sensor Networks (WSNs). Businesses, industries, and the military have utilized WSN to track an object or monitor a phenomenon. Besides, many types of research areas have emerged from the WSNs domain such as from routing protocol, security, and privacy to data mining and many others. Nevertheless, currently, researchers are concerned with improving the performance of the WSNs technologies [2]. In WSN, sensor nodes are equipped with sensing, processing, radio, and power unit, yet they have limited resources in terms of energy, computation, and storage [3]. Frequently, a large number of the sensor nodes are deployed widely in the target environment and continuously communicate the phenomenon measurement like ambient temperature, relative humidity, soil moisture, and wind speed to the base station. Therefore, in most situations, sensor data need to collect accurate and reliable measurements for data analysis and decision-making especially in a critical domain such as in meteorology stations, military applications as well as security monitoring. Unfortunately, the raw data collected from WSN communications usually are not reliable and inaccurate due to the imperfect nature of WSNs [2]. The reason is that the sensor nodes are deployed in a harsh and unattended environment, and vulnerable to malicious attacks. Therefore, data collected from these sensor nodes are often generates missing data, duplicated or error records. To ensure the collected data is reliable and accurate for data analysis and decision-making, one of the solutions is to detect erroneous data, malicious attacks, or changes in the environment namely anomaly or outlier detection (These terms will be used inter changeably throughout this paper). Anomaly detection is one of the potential approaches that can be considered as a solution. Furthermore, it is defined by [4] as the process of identifying data patterns that vary from anticipated behaviour. When it comes to WSNs, anomaly detection has been widely employed across a wide range of industries such as the military and environmental sectors [5]. This is due to the characteristic of low-cost, small in size, and multi-functional sensor nodes; it helps to achieve the need for fast and cheap data collection. 

Identifying misbehaviour or anomalies in the network is important in providing liable and secure functioning of the network [6]. In comparison to networks like MANET and BAN, sensor network has resource constraint and has been a major designing challenge in lightweight detection scheme. Although many techniques have been proposed to design and develop a lightweight anomaly detection model for WSNs, effective and efficient solutions are still a major research challenge. The common design issue in the existing solution lay in the online updating of the detection model and the use of communication overhead among sensors nodes.

This paper focuses on designing a lightweight anomaly detection scheme to provide reliable data collection while consuming less energy using one-class learning schemes and dimension reduction concepts. This paper aims to design and develop a lightweight anomaly detection by accurately detecting anomalous data while utilizing energy efficiently. One-class support vector machine (OCSVM) is used as an anomaly detection algorithm due to its advantage in classifying unlabelled data while the hyper-ellipsoid variance can detect multivariate data. Various OCSVM formulations have been proposed such as a hyper-plane, hyper-sphere, Quarter-sphere, Hyper-Ellipsoid, and Centred-Ellipsoid (CESVM). On the other hand, to decrease the computational complexity, CCIPCA is utilized to reduce data dimensions.

An accurate sensor measurement is needed to make an important decision at the end- point such as the base station. However, the raw data collected from sensors may be inaccurate due to many reasons. For instance, hardware failures, changes in the environment as well as malicious attacks that may produce anomalies or outliers in data. These data anomalies or outliers can be detected by designing and developing an anomaly detection model. Unfortunately, large data distribution in WSN makes communication overhead and needs more computational complexity as well as increases memory usage thus reducing the efficiency of the anomaly detection model [7,8]. Furthermore, the enormous volume of data acquired from sensor nodes contains irrelevant and redundant features [9,10], resulting in increased resource usage and a decrease in detection effectiveness Moreover, multivariate data is also needed to be considered when designing an anomaly detection scheme as multivariate data are always sensed in the target phenomenon [11,12] which also contributed to the energy exhausted. Due to these factors, therefore, lightweight anomaly detection which incorporated dimensionality reduction is crucial as proposed in [13,14].

Many anomaly detection solutions such as in [4,15,16,17,18] specifically in WSNs, use a one-class classifier, such as the One-class Support Vector Machine (OCSVM), to construct the normal reference model. The limitation of these solutions is the classification over high-dimensional data which increases the computational complexity and the communication overhead. Meanwhile, the unsupervised Principal Component Analysis (UNPCA) based solutions such as in [7,19] are more lightweight and efficient due to the dimension’s reduction used before the classification. However, the effectiveness in terms of the detection accuracy of such solutions needs to be improved. Therefore, a lightweight and effective anomaly detection that incorporated dimensionality reduction with an effective classifier is needed. To this end, in this study, a lightweight and effective anomaly detection scheme is proposed. The contribution of this study can be summarized as follows:

1. An effective and efficient anomaly detection scheme is designed and developed by combining the unsupervised One-Class Support Vector Machine (OCSVM) with the Candid Covariance-Free Incremental Principal Component Analysis (CCIPCA) to decrease the computational complexity and improve memory utilization while increasing the detection accuracy.
2. Various OCSVM formulations have been investigated such as a hyper-plane, hyper-sphere, Quarter-sphere, Hyper-Ellipsoid, and Centred-Ellipsoid (CESVM) to improve the detection accuracy for multivariate data.
3. The Candid Covariance-Free Incremental Principal Component Analysis (CCIPCA) has been incorporated in the design to reduce the data dimension and thus decrease the computational complexity and improve memory utilization.
4. Extensive experiments have been conducted to evaluate and validate the effectiveness and efficiency of the proposed scheme.

The rest of this paper is organized as follows. Section 2 presents the related work. Section 3 describes the proposed CESVM-DR Scheme in detail while Section 4 explains the experimental design and setup. Results and Discussion are presented in Section 5 and this paper is concluded in Section 6.

## 2. Related Work

The quality of data sensed by sensor nodes is a crucial issue in WSNs. Erroneous and malicious data can affect data quality [20]. Some of these anomalous data may come from faulty nodes. These erroneous or malicious data are usually known as anomalies or outliers. Outliers in data collected by WSNs can be caused by a various factors, including noise and error, real events, and malicious activities [21]. Therefore, anomaly detection is implemented in WSN to provide more accurate data collection for further analysis at the base station. Nevertheless, accurate and reliable data are needed for critical decision-making such as disaster, or fraud detection. Furthermore, assuring the security of wireless sensor networks and preventing malicious attacks requires the ability to identify anomalous behaviour [22].

The anomaly detection techniques learn normal behaviour and construct a model for normal events in the network [23]. The anomaly detection techniques are either based on the type of background information about the data presented [24,25] or by the type of model they learn. The first approach categorizes anomaly detection into supervised, unsupervised, and semi-supervised. The classifier is trained using labelled data in a supervised mechanism to identify anomalous data. Meanwhile, the unsupervised mechanism, identifies anomalous data without any prior understanding of the data. Lastly, to define a normality boundary, a semi-supervised learning technique uses training on pre-labelled normal results. The second approach categorizes the taxonomy of anomaly detection into classification-based, nearest-neighbour-based, clustering-based, statistical-based, information-theoretic, and spectral-based anomaly detection techniques in [4]. The same taxonomy of anomaly detection techniques is found in the latest survey by [23,26].

The design of anomaly detection model has been addressed using several techniques. used to construct the anomaly detection model. Among these techniques, the one-class classifier is preferred in the WSNs due unsupervised approach [15,27,28,29,30] can be utilized in the absence of a ground truth labelled dataset. One-class classifiers have evolved as a method for scenarios in which only one of two classes in a two-class issue has labelled data [31]. The fundamental concept of OCSVM is to use the feature space’s origin as a representation of the abnormal data and then isolate the target sample from the origin by the maximum possible margin [32]. Moreover, anomalous data may often be insufficient due to difficult acquisition or costly manual labelling, thus one-class learning is usually favoured. One-class classifiers are categorized under unsupervised learning by having all the data objects with the same label in the target class. A few anomaly detection solutions, specifically in WSNs, use a one-class classifier to construct the normal reference model, such as the One-class Support Vector Machine (OCSVM) [4,15,16,17,18]. The limitation of these solutions is the classification over high-dimensional data which increases the computational complexity while increasing the communication overhead. While anomaly detection solutions such as in [7,19] use the unsupervised Principal Component Analysis (UNPCA) to construct the reference model which is more lightweight and efficient due to the dimension reduction approach used in the UNPCA. However, the effectiveness in terms of the detection accuracy of such solutions needs to be improved.

Feature space mapping is the core feature of one-class SVM-based outlier detection approaches. Data vectors obtained in the original space are mapped into feature space in a higher dimensional space. In the resulting feature space, a normal data decision boundary is determined that contains most of the data vectors. An outlier is a data vector that is beyond the boundary [33]. Several types of OCSVMs were formulated which can be distinguished by their shapes and formulations namely Hyper-plane [34], Hyper-sphere [35], Quarter-sphere (QS-SVM) [36], Hyper-ellipsoidal (TOCC) [37] and centred ellipsoidal (CESVM) [15]. A study by [38] indicates that the generalization capability and classification performance of OCSVM formulations can be ranked in decreasing order as follows:

Hyperellipsoid ≈ Centred Ellipsoid > Hypersphere ≈ Quarter-Sphere > Hyperplane

On the other hand, data transmission is the main cause for energy depletion com-pared to the data sensing and processing by the sensor nodes. Commonly, there are two ways the sensing data impacts the energy consumption includes the unneeded data sample and power consumption of the sensing subsystem. For its ability to cope with enormous amounts of data, dimensionality reduction has been highlighted as an effective 203 method to overcome the “curse of dimensionality” [39]. Therefore, dimensional reduction is performed to reduce the amount of data sensing data while maintaining sufficient data 205 quality or accuracy after data is transferred to the base station. Moreover, the dimension reduction as the energy-efficient mechanism is one approach to obtain a reduction in the number of data to be sent to the sink [40]. The dimensional reduction schemes based on PCA and its variants, DWT, and ANN algorithm for instance have been used to encode high dimensional data to low dimensional data.

Dimensional reduction techniques have been discussed in [39,40,41,42,43]. Due to computational and energy restrictions in sensor nodes, developing a dimensionality reduction model for WSNs that is suitable to this constraint domain is necessary. Besides, many studies have been developed in univariate settings while few multivariate dimensional reduction models have been introduced. PCA-based dimensional reduction in a univariate data is proposed in [44,45] where PC computing is distributed to sensor nodes. In [44], the network architecture is based on clustered structure and data is collected from the first network layer, while the rest of the layers will perform dimension reduction using CA-based dimension reduction algorithm. Meanwhile, Ref. [45] is based on aggregation tree structure in two-hops communication between sensor node, an intermediate node, and base station. Ref. [19] used kernel PCA (KPCA) to classify outliers data in WSNs as well as to reduce data dimension. Since PCA is one of the encoding algorithms, in this research, KPCA is used in pre-processing step to extract significant features using Mahalanobis kernel thus reducing the data size before the distance-based anomaly detection is applied. The real-world environment consists of multivariate data, thus designing the multivariate dimension reduction is essential to address the power and memory constraint of the WSNs. Few multivariate data reduction models have been proposed in [46,47,48,49,50]. PCA is a popular multivariate data analysis method used to reduce the dimensionality of a set of correlated data observations into a set of uncorrelated variables known as principal components (PCs) [8,51].

The primary purpose of this study is to design more energy-efficient communication in WSNs and reduce the computational complexity. Besides the encoded data accuracy must be maintained after the data is decoded on the recipient’s side. Therefore, choosing the lightweight dimension reduction algorithm can contribute to energy-efficient communication, low computational complexity, and may also improve data accuracy. Candid Covariance-Free Incremental Principal Component Analysis (CCIPCA) data dimension schemes based on PCA have been proposed by [7] for the WSNs domain. The CCIPCA algorithm was introduced by [52] to tackle the issue of high-dimensional image vectors. This paper addresses energy-efficient WSN by proposing the CCIPCA reduction technique. Meanwhile, designing one-class support vector machine-based anomaly detection can address the issues of unlabelled data due to the absence of ground truth labelled dataset and a costly manual labelling. Furthermore, using centred ellipsoidal (CESVM) as a one-class classifier results in a considerable reduction in complexity owing to the linear optimization problem, which is considerably less expensive [38]. Therefore, the proposed lightweight anomaly detection scheme in this study combines the CCIPCA with the unsupervised OCSVM based CESVM kernel to decrease the computational complexity, improve memory utilization, and increase detection accuracy.

## 3. The Proposed CESVM-DR Scheme

**Figure 1:**
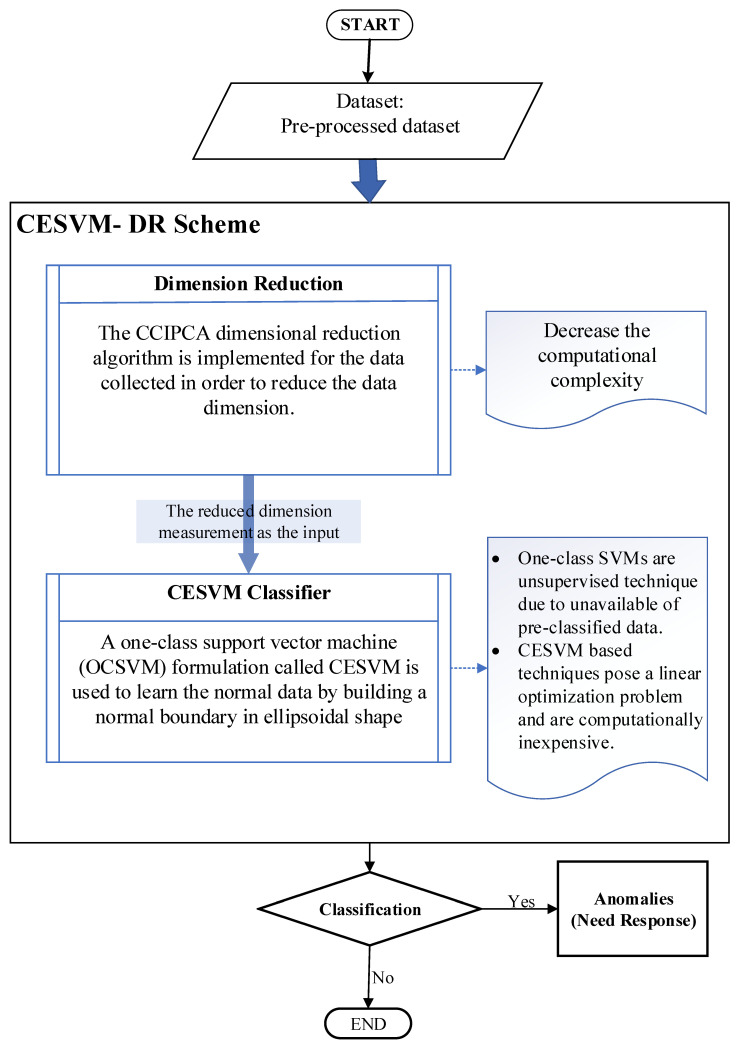
The Overview of Proposed CESVM-DR anomaly detection scheme.

In this section, the proposed CESVM-DR scheme is described in detail. The proposed CESVM-DR is a lightweight anomaly detection scheme. CESVM-DR incorporates the dimension reduction method into the one-class anomaly detection to minimize the communication overhead, computational complexity as well as memory utilization. By implementing dimension reduction in the scheme, less energy will be consumed in the sensor nodes due to its’ reduced data size. On the other hand, the proposed anomaly detection scheme is developed to detect anomalies based on the one class learning scheme namely One-Class Support Vector Machine (OCSVM) technique. One-class classifiers are unsupervised techniques because they only require input data samples without their labelling into normal or abnormal samples. As stated by [49], the one-class classifies concepts that learn the data during data training without requiring the labelled data. The one-class unsupervised techniques learn the boundary around normal instances during training while some anomalous instances may exist and declare any new instance lying outside this boundary as an outlier. Due to the pre-labelled data being difficult to obtain in WSNs environment, thus CESVM formulation which is a one-class setting is a suitable anomaly detection approach in the WSNs domain. The proposed CESVM-DR detection scheme contains two phases: training and detection phase, as shown in Figure 1. The first phase is conducted offline meanwhile the testing phase is conducted online to classify the new data measurement into normal or anomalous data measurements. Further explanation of the training and detecting phase of the proposed CESVM-DR will be conducted in the next section.

### 3.1. Description of Proposed CESVM-DR Scheme

**Figure 2:**
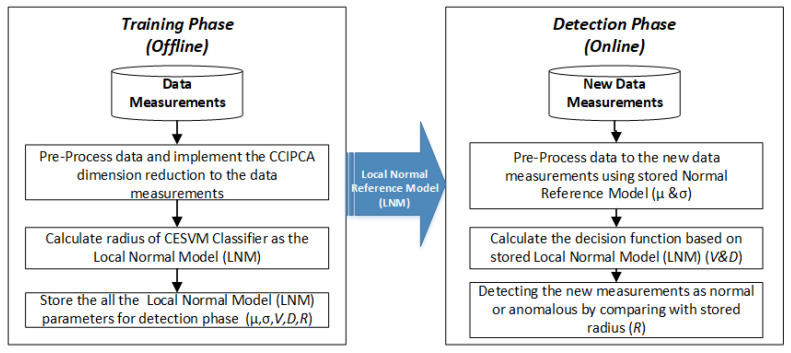
The training and detection phase of the proposed CESVM-DR anomaly detection scheme.

This section described the proposed CESVM-DR anomaly detection scheme which consists of the training and the detection phase as shown in Figure 2. The first stage is used to learn the normal behaviour of sensor data at every sensor node in offline mode. Ref. [53] stated that to improve the CESVM scheme with a low-rank representation of the gram matrix for use in distributed detection algorithms with lower communication overhead. Therefore, the proposed anomaly detection classifier adapts a dimension reduction method to reduce the data dimension. It is carried out first by selecting data observations with the size of m to obtain the centered-ellipsoid effective radius R in a specific period. This method of collecting the size of data observations in a specific period has been adapted in several online anomaly detection schemes such as [52,54,55,56,57]. After the data measurement is standardized by mean and standard deviation, CCIPCA is applied to reduce the dimension of the data measurements. Then, the effective radii R will be calculated using Equation (1). All the computed parameters which are mean (µ), standard deviations (σ), Eigenvector (V), and Eigenvalue (D) are stored at each node to be used in the next phase which is detection phase.

(1) $$Md\;\left(x\right)=\|\sqrt{m}{\;\Lambda }^{-1}{\;Ρ}^{Τ}{\;Κ}_{c}^{i}\|$$

#### 3.1.1. Training Phase (Offline)

**Figure 3:**
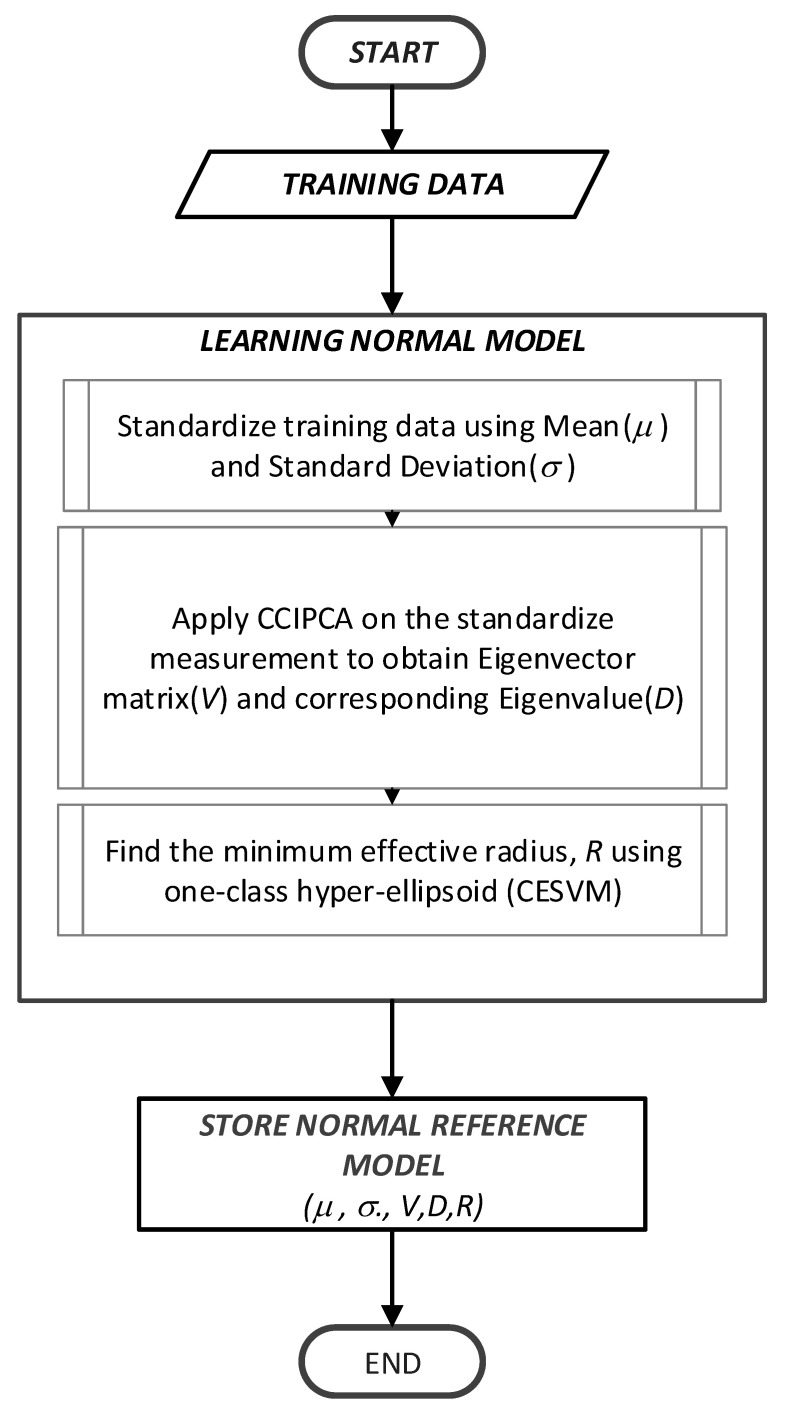
Flowchart of Training Phase for the CESVM-DR Model.

As mentioned earlier, CESVM is used as a classifier to build the normal reference model in this proposed anomaly detection scheme. The nature of an ellipse is based on the covariance of the dataset, thus CESVM is suitable for sensor data where multivariate attributes may induce a certain correlation. For instance, the readings of humidity sensors are correlated to the readings of temperature sensors. In addition, computational complexity, especially computing of reduced eigenvalue and eigenvector for radius calculation in ellipsoidal CESVM motivates the design of lightweight detection techniques. In this study, dimension reduction technique namely CCIPCA proposed by [54] as the solution to obtain both eigenvalue and eigenvector to be used in anomaly detection model. The proposed model, CESVM-DR, is illustrated in Figure 3.

The procedures of the training phase are described as follows:

1. Raw data measurements are collected at each of the sensor nodes to build the normal reference model.
2. The collected data are standardized using mean, µ and standard deviations, σ using mean-centered value.
3. The dimension reduction based on the CCIPCA algorithm is applied to reduce the data dimension and a suitable number of the principal component is chosen.
4. The minimum effective radius, R is calculated based on a calculation of R = $\left\|\sqrt{m}\;\Lambda^{-1}\;{Ρ}^{Τ}\;{Κ}_{c}^{i}\right\|$. This parameter is known as the normal reference model to be used in the detection phase.
5. All calculated parameters including σ, µ, V, D, and R are stored in the node. The Eigenvector (V) and Eigenvalue (D) are calculations taken from calculating the centered kernel matrix of the training data.

At the sensor node, all the parameters calculated in the training phase are regarded as the normal state. They will be used to build the normal reference model. The normal reference model is constructed offline, similar to the methods of [7,15,51,57]. The detailed pseudocode algorithm for the training phase of this scheme is shown in Algorithm 1.
**Algorithm 1:** Pseudocode algorithm for the training phase of proposed CESVM-DR Scheme**Input:**$S_{Test}$ // a matrix (n×m) of sensor measurements collected from specific time period **Output:** are mean (µ), standard deviations (σ), Eigenvector (V) Eigenvalue (D) and minimum effective radius (R)
1.**Do** the following step:
2.Standardize $S_{Test}$ using mean (µ), standard deviations (σ)3.Apply CCIPCA on Standardize $S_{\mathrm{Train}}$ to obtain Eigenvector (V) Eigenvalue (D)4.Calculate minimum effective radius (R):$R=\|\sqrt{m}\;\Lambda^{-1}\;{Ρ}^{Τ}\;{Κ}_{c}^{i}\|$5.Store the normal reference model (µ ,σ , V, D, R) to be used on detection phase6.**End**


#### 3.1.2. Detection Phase (Online)

For the detection phase, new data measurement will be detected as normal or anomalous. Using the stored reference model obtained from the training phase, the decision function is calculated as shown in Equation (2).

(2) f (x)=sgn(max(R−Md(x))

**Figure 4:**
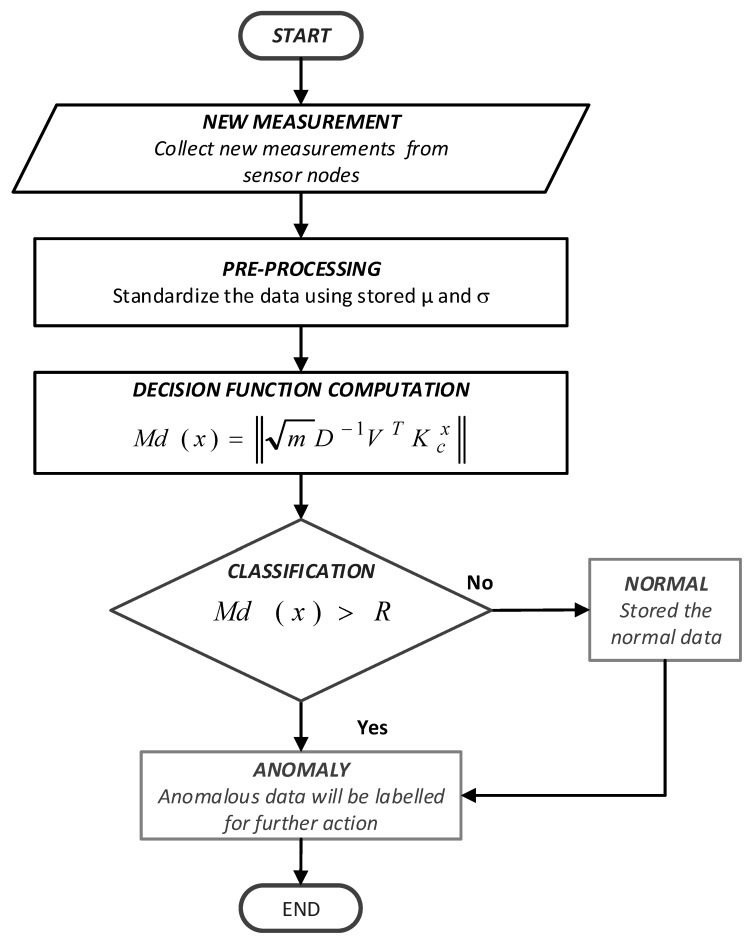
Flowchart for Detection Phase in the proposed CESVM-DR Scheme.

From this decision, the new measurement will be classified as anomalous if the reading is negative and vice versa where V and D are equivalent to P and Λ. Negative results indicate that the reading is larger than effective radii, R which represents the normal behaviour of training data thus the data is classified as anomalous. Figure 4 shows the flowchart of the detection phase of the proposed CESVM-DR anomaly detection scheme. The pseudo-code algorithm for the detecting phase of the proposed CESVM-DR scheme shows in Algorithm 2.
**Algorithm 2:** Pseudocode algorithm for the detection phase of proposed CESVM-DR Scheme**Input:**$S_{Test},µ,\sigma ,V,D$ and R // $S_{Test}\;a\;matrix\;\left(n\times m\right)$ of real time sensor measurements
**Output:** Normal or Anomalous type of class WSN data
1.***Do*** the following step:
2.Standardize $S_{Test}$ using same mean (µ), standard deviations (σ) from training phase3.Calculate decision function f (x):     f (x) = sgn(max(R−Md(x))4.Compared the decision function f (x) value with R calculated in training phase to class the measurement as normal or anomalous as following condition:
     *If*
f (x) > R
*then*
         *Class* = *Anomaly*
     *Else*,
      *Class = Normal*
5.***End***


The procedure of the testing phase is described as follow:

1. New data observations of m size collected from the sensor nodes are standardized to the calculation in the training phase using mean (µ) and standard deviation (σ) of the normal reference model.
2. The distance of each new measurement is calculated as described in Equation (1), using the stored normal reference parameters which are Eigenvector (V) and Eigenvalue (D). The measure similarity between data is based on decision function
3. These new data measurements are classified as normal or anomalous using the decision function in Equation (2).

The new data measurements are classified as anomalous if the decision function is indicated as negative, meaning their distances from the center is larger than the distances of the normal reference model. Otherwise, it is normal.

In the proposed lightweight CESVM-DR anomaly detection scheme, the complexity is reduced by incorporating the CCIPCA algorithm to minimize the computation complexity of the CESVM algorithm. Therefore, the high communication cost due to the broadcast of the entire covariance matrix among the nodes of the network is reduced by applying CCIPCA to the proposed CESVM-DR scheme especially dealing with multivariate data. To evaluate the detection effectiveness, the performance measures which are represented by detection rate (DR), detection accuracy, and false alarm rate (FNR and FPR) are analysed. These performance measures are used in other anomaly detection models including in [7,15,19]. Meanwhile, the computational complexity and memory utilization, and communication overhead are used to evaluate detection efficiency.

## 4. Experimental Design

This section explains the process of experiment setup including preprocessing and data labelling. Data labelling techniques used to label the unlabeled data as well as the data processing procedure will also be discussed in this section.

### 4.1. Preprocessing

Before data labelling, raw data has been undergone the pre-processing process. Raw data collected from sensor nodes is standardized to transform into a standard format using the mean and standard deviation. During the standardization process, the observation data are centered to avoid biased estimation as mentioned in the previous section. The new standardized data can be calculated as illustrated by Equation (3).

(3) $$x_{new}=\frac{x-\mu }{\sigma }$$

Every data measurement in the training dataset is standardized using Equation (3). The parameters *µ* and *σ* are the mean and standard deviation of the training dataset. Both *µ* and *σ* are stored in the sensor nodes and will be used to standardize the new data measurements of the testing dataset during the online detection. The CCIPCA dimension reduction scheme will then be applied to the data instances to reduce data dimensionality. The reduced data will then be used as the input for the CESVM classifier.

### 4.2. Datasets and Data Labeling

The datasets used to evaluate the proposed CESVM-DR detection scheme are obtained from GSB, IBRL, LUCE, PDG, and NAMOS datasets. These datasets have been used in several WSN researches included in several WSN researches included in [7,14,15,19,28] GSB dataset was labelled using simulated-based labelling technique while histogram labelling technique was applied to IBRL, LUCE, PDG, and NAMOS dataset.

Similar to the study in [57], simulated-based labelling was created in this paper. The simulated-based labelling approach is used to label the data measurements taken from a small cluster of GSB sensor deployment. Data measurements were extracted from nodes N25, N28, N29, N31, and N32 are named D1, D2, D3, D4, and D5 respectively. For each dataset, artificial anomalies were randomly generated. The statistical properties of the normal data, such as mean and standard deviation, are computed and used to construct artificial anomalies. The statistical characteristics measured by the mean and standard deviation of both normal and generated artificial anomalies for datasets are presented in Table 1 (Ambient temperature and relative humidity are selected as an example). Following the study [57], artificial anomalies were created randomly using statistical parameters (see Table 1) that differ slightly from the properties of the normal data based on these properties. The artificial anomalies generated randomly based on the statistical properties were injected into the normal data to be used in the experiments.

The artificial anomalies based simulated-based labeling approach is generated as follows:

1. The normal data measurements are collected from a real-life dataset with the size of m×n where 𝑚 and 𝑛 represent the number of data measurements and the number of variables respectively. The mean µi and standard deviation σi of the collected data measurements are calculated with i=1, 2,…, n.
2. The new *µ* and σ values to generate artificial anomalies are selected by adding µi and σi with the preferred amount of deviation to produce slightly different mean and standard deviation from normal values.
3. Based on the selected new µ and σ values, the artificial anomalies are produced based on normal random distribution function, *f* as follow: *Artificial* *anomalies* = *f* ( *µ*, *σ*, *m*, *n*).

**Figure 5:**
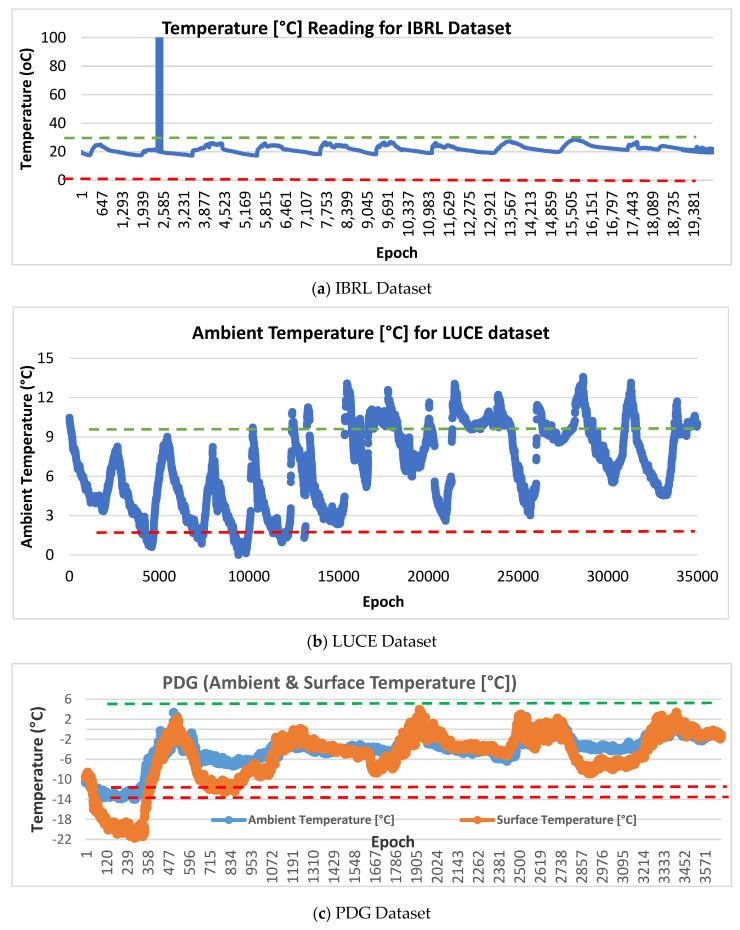

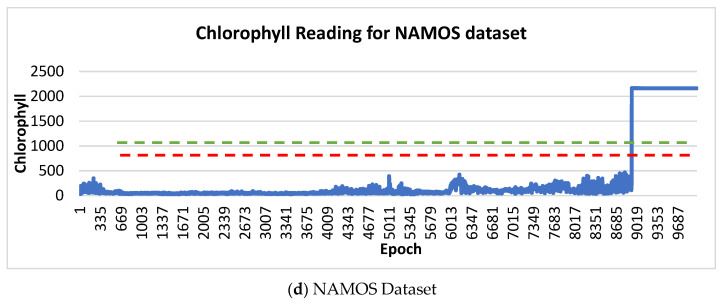
Histogram plots for (**a**) IBRL, (**b**) LUCE, (**c**) PDG and (**d**) NAMOS Dataset.

The experiments have been conducted with histogram-based data labelling using IBRL, LUCE, PDG, and NAMOS datasets. In the histogram-based labelling approach, datasets are plotted using histogram-based, and anomalous data instances are labelled with visual inspection based on the normality regions of the dataset as in Figure 5. This labelling technique has been used in [7,51,58,59,60,61] to label the unlabeled dataset. From visual investigation on Figure 5, different scenarios on selecting training and testing size are investigated to measure the performance of detection effectiveness of proposed CESVM-DR. The detailed performance result for each dataset is discussed in the next sections.

### 4.3. Testing Procedures

The experiments were conducted in two stages. A regularization parameter (v) was tested in the first stage to determine the probability of outliers in the data. In general, a high detection rate resulted when the parameter v is set to a large value, yet this led to a greater false alarm rate [16]. As the number of outliers is unknown in the dataset, hence, technique’s robustness is assessed utilizing parameter *v*. The robustness performance can be determined, regardless of the v value as the detection rate reading is higher while the false alarm rate is low. For this experiment, the value of v is varied between 0.05 and 0.1 which indicates the value of outliers from 5% to 10%. Meanwhile, the test use three kernel functions as follow:

1. Linear function:

(4) kLinear(x1, x2)=(x1·x2)

2. Radial basis function (RBF)

(5) $$k\;\left(RBF\right)\;\left(x1,\;x2\right)=exp({\left\|x1-x2\right\|}^{2}/2\sigma^{2})$$

where σ is the width of the kernel function.

3. Polynomial function

(6) $$kPoly\;\left(x1,\;x2\right)={\left(x1\cdot x2+1\right)}^{r}\;$$

where r is the width of the kernel function.

Based on the results obtained, the best kernel function with an acceptable parameter *v* among the three will be used in the second stage of the experiment. 250 normal measurements were selected from the GSB dataset in 10 runs while 10 percent (10%) artificial anomalies were randomly generated based on the simulated-based.

### 4.4. Performance Evaluation

In this experiment, the proposed CESVM-DR scheme was evaluated in terms of effectiveness and efficiency. The detection effectiveness is analyzed in terms of the detection rate (DR), detection accuracy, false-positive rate (FPR), and false-negative rate (FNR) of the datasets mentioned. Meanwhile, the efficiency is analyzed by measuring the computational complexity and memory utilization, and communication overhead. The effectiveness of the proposed CESVM-DR scheme is evaluated using both simulated-based and histogram-based labelling types while the efficiency is evaluated by analyzing the big O notations tested schemes.

For evaluation, the proposed CESVM-DR scheme is compared with other related CESVM anomaly detection schemes using simulated-based labelling. As the core anomaly detection classifier is adopted from CESVM [15] it is used as a benchmark against the proposed CESVM-DR scheme. In addition, the proposed CESVM-DR is compared with the EOOD scheme [16] and (kPCA) [19]. The evaluation is examined using the RBF kernel function with the kernel width of 2 because it is the most stable kernel width among many experimented values in this study.

## 5. Results and Analysis

The proposed CESVM-DR scheme is evaluated by comparing its performance in terms of the effectiveness (DR, FPR, FNR, and Accuracy) and efficiency (Big *O* notations of Computational Complexity, Memory Utilization, and Communication Overhead) with the related work using both datasets, the simulated-based and histogram-based labelling types as follows.

### 5.1. Effectiveness Evaluation

The effectiveness evaluation has been conducted by evaluating the proposed scheme with the related work using both datasets, the simulated-based and histogram-based labelling types as follows.

#### 5.1.1. Accuracy Evaluation Using the Simulated-Based Data Labelling

**Figure 6:**
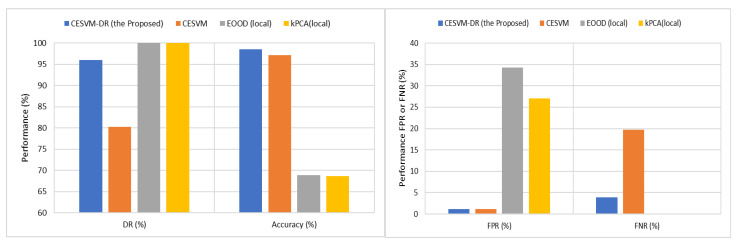
Average Performance Comparisons Accuracy and DR (**left**) while FPR and FNR (**right**).

The proposed CESVM-DR scheme is compared with other related CESVM anomaly detection schemes using simulated-based labelling. As the core anomaly detection classifier is adopted from CESVM [15] it is used to be the benchmark against the proposed CESVM-DR scheme. Meanwhile, another related CESVM anomaly detection scheme proposed by [28] called the EOOD scheme is used to evaluate the proposed scheme. The latest anomaly detection model based on kernel PCA (kPCA) was introduced by [19] another anomaly detection scheme used to validate the experimental results. The evaluation is examined using the RBF kernel function with a kernel width of 2 as the most stable kernel width results in the previous section. Figure 6 summarizes the average of the accuracy performance (effectiveness) of the tested scheme compared with the proposed CESVM-DR scheme while Table 2 presents the results in detail.

As shown in Figure 6 and Table 2, the proposed CESVM-DR scheme outperforms all of the CESVM schemes in all experiments while maintaining a stable 1.2% FPR and 3.92% FNR. On the other hand, EOOD (local) and kPCA (local) outperform some of the datasets compared to CESVM-DR in terms of detection rate and FNR. However, due to higher FPR reported in the EOOD (local) scheme, affects the detection accuracy as compared to the CESVM-DR scheme. It indicates that both the CESVM schemes and kPCA used the same Mahalanobis distance in the algorithm to classify the anomalous data. This distance measure considers the attribute correlation thus can be useful to detect multivariate datasets. As CESVM-DR, CESVM and EOOD are based on the ellipsoidal formulation that also considers multivariate and attributes correlations which separated the normal and abnormal class within the ellipse geometric formulation. Meanwhile, the proposed CESVM-DR using CCIPCA to produce the eigenvector matrix and their corresponding eigenvalues give the advantage to the performance measure results. EOOD on the other hand, model hyper-ellipsoid SVM in the input space and fix the center of hyperellipsoid at the origin. Meanwhile, kPCA is using PCA to detect the anomalous is the only scheme not based on the CESVM algorithm. Therefore, this scheme has no parameters to be tuned.

A test of significance, namely a *t*-test, was used to evaluate the statistically significant difference of accuracy, DR, FPR, and FNR between CESVM-DR with CESVM, EOOD (local), and kPCA (local) anomaly detection schemes. The results show that the difference between the proposed and all tested schemes are significant with all studied performance measures across all the datasets in favour of the proposed CESVM-DR scheme except with DR of EOOD (Local) and kPCA schemes with datasets D4 and D5. However, EOOD (Local) and kPCA schemes failed to strike a balance between FPR and DR which is achieved with significance by the proposed CESVM-DR scheme.

#### 5.1.2. Accuracy Performance Result Using Histogram-Based Dataset

**Figure 7:**
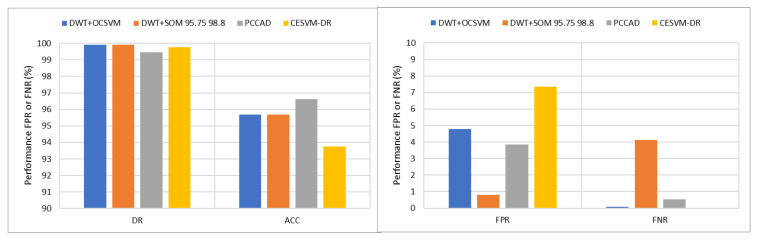
Average Performance Comparisons Accuracy and DR (**left**) while FPR and FNR (**right**).

The proposed CESVM-DR scheme was benchmarked against other related anomaly detection schemes in PCCAD [31], DWT + SOM [59], and DWT + OCSVM [58] as the same histogram-based data samples were used in these researches. The performance evaluation is presented in Figure 7 and Table 3.

From Table 3, considering the proposed CESVM-DR, the detection rate of 100% and the false-negative rate of 0% are reported in both IBRL and NAMOS datasets. This is due to both datasets were containing univariate data features. Meanwhile, as illustrated in Figure 5a,b short and constant anomalies are present in IBRL and NAMOS datasets respectively, thus the proposed anomaly detection schemes successfully reported better results compared to the PDG dataset in which noise anomalies were presented. For IBRL datasets, the false positive rate of the proposed CESVM-DR is slightly higher than DWT + SOM and PCCAD. On the other hand, DWT + OCSVM and the proposed CESVM-DR schemes are OCSVM based classifiers that result in slightly high FPR. In contrast, the proposed CESVM-DR schemes outperformed all three detection schemes when the NAMOS dataset is used. Meanwhile, varying results are obtained in the PDG dataset for all schemes. Again, DWT + OCSVM and the proposed CESVM-DR scheme reported higher results in terms of detection rate and false-negative rate compared to DWT + SOM and PCCAD schemes.

However, the proposed CESVM-DR scheme reported a high false-positive rate and low detection accuracy compared to other schemes. The false-positive rate is mainly concerned as it represents the normal data which is classified as anomalous data thus affecting the accuracy performance. The datasets PDG exhibited the highest false-positive rate as compared to other datasets. As the PDG dataset is taken from Patrouille des Glaciers the outdoor and extreme environment dataset shows more dynamic datasets compared to other datasets. As shown in Figure 5c, which illustrates measurements from the PDG dataset, the noise measurements are very similar to the normal measurements. Such noises affect the performance of anomaly detection. Furthermore, the dynamic data changes require the tuning of such parameters to cope with these changes. Other reasons include that the cause of the reading used in the training dataset does not reflect the current situation of the new measurement during the detection is performed. Moreover, the normal reference model must be selected carefully in order which describe the overall behavioural data. Therefore, the normal reference model must be updated from time to time to ensure the effective results of the detection performance and false alarm rate.

### 5.2. Efficiency Evaluation

The efficiency of the anomaly detection model can be measured based on memory utilization, computational complexity, and communication overhead. The efficiency evaluation of the proposed CESVM-DR is compared with CESVM, EOOD, PCCAD, kPCA, DWT + SVM, and DWT + SOM schemes the same as a comparison in effectiveness evaluation. The efficiency evaluation will be evaluated in Big O notation. The efficiency evaluation results are shown in Table 4 and the description of the parameters used are listed in Table 5.

From Table 4 and Table 5, the proposed CESVM-DR is more efficient due to the few factors that are included with the linear optimization compared with the baseline CESVM. That is, the memory complexity CESVM-DR scheme is O(mn+nd) where d<p and d represent the reduced dimension of the data vector and m<d. Thus, O (mn+nd) is equivalent to the O(nd). With regards to computational complexity, the proposed CESVM-DR is formulated as a linear optimization problem, then it is more feasible for implementation on WSNs [38]. That is, the dimensional reduction using CCIPCA added the feasibility of the proposed scheme for implementation on WSNs. In terms of the communication overhead, the proposed CESVM-DR scheme doesn’t require any communication to perform the anomaly detection and thus, it is efficient in terms of energy consumption, which is the common design issue for anomaly detection in WSN. The following subsections present a detailed analysis of the efficiency evaluation.

#### 5.2.1. Memory Utilization

The proposed CESVM-DR operates in two phases: training and testing. The computation is performed during the training phase, which comprises data processing utilizing mean (μ) and standard deviation (σ) parameters, and is kept to be used in the subsequent phase. Then, Eigenvalue (Λ) and the corresponding Eigenvector (P) which are calculated using CCIPCA are also stored in the sensor. Radius, R is calculated to determine the cen- tered hyper-ellipsoid border based on calculated eigenvalue (Λ) and corresponding ei- genvector (P).

For memory utilization, the baseline CESVM keeps the eigenvalues, P, and eigenvectors, Λ after the linear optimization is done, thus the complexity is represented by O(mn+np). In the calculation, m represents a low-rank approximation of Kernel Gram of RBF kernel function, data observation amounts are denoted by n and p is the data vector’s dimension, where p<n. Furthermore, the memory complexity of the proposed CESVM-DR is O(mn+nd) where d<p and d represent the reduced dimension of the data vector. The EOOD system is primarily concerned with storing in the memory the observations of the sliding window’s size as O(np). Due to the assumption that n>p, storage overhead for other parameters like the covariance matrix, which has a cost of $O\left(p^{2}\right)$, is insignificant. The PCCAD does not involve any Kernel Gram calculation, therefore, the memory utilization is only O(nd). As the other schemes operate at the base station and sensor nodes are involved in collecting the data, therefore only O(𝑛) of memory is utilized. Meanwhile, kPCA yet involved Mahalanobis kernel thus memory utilization represents as O(k+np) where k represents as Mahalanobis kernel.

The memory utilization in the proposed CESVM-DR is driven from baseline CESVM while the reduced dimension has been applied using CCIPCA which has less memory usage. Because CESVM is formulated using linear optimization, it only requires the de-termination of a radius and this gives benefits to memory utilization. PCCAD used CCIPCA to reduce the data dimension as our proposed CESVM-DR which also reduced memory utilization. Meanwhile, no dimension reduction is involved in both EOOD and kPCA.

#### 5.2.2. Computational Complexity

As mentioned earlier, the proposed CESVM-DR operates in the training and testing phase where the training phase is done in an offline manner while the testing phase is done in an online manner. Therefore, the computational complexity of the CESVM-DR is evaluated in the testing phase only. During the testing phase, the calculation is done by (1) standardize the new data measurements using stored (μ) and (σ) parameter; (2) calculate the distance measure (Md(x)) for every new data measurement based on stored Λ and P; (3) compare the distance measured with the radius R to classify the new measurement as either normal or anomalous.

As the calculation of CCIPCA, O(N) is done in the training phase where N is several variables, therefore a total computational complexity of CESVM-DR is $O\left(N\left(m^{2}d\right)+dn^{2}\right)$ which $O\left(n^{2}\right)$ represents a kernel matrix (also called a Gram matrix) and d represents the reduced dimension of the data vector. Meanwhile, the computational complexity of CESVM scheme involves the computation of a kernel matrix and the complexity of calculation the Eigen-decomposition is $O\left(n^{3}\right)$. However, the eigenvalues decay exponentially for a wide variety of kernels such as the RBF. Therefore, the total computational complexity of CESVM is represented as $O\left(n^{2}+m^{2}n\right)$ where m<n that represents the low-rank approximation of the kernel Gram matrix. On the other hand, the computational complexity of EOOD mainly depends on solving a linear optimization problem, which is represented as O(P), as well as computing covariance matrix, which is represented as $O\left(mp^{2}\right)$. Meanwhile, computational complexity in kPCA, involved the calculation of PCs with a complexity of $O\left(np^{2}\right)$.

Meanwhile, as the calculation of PCCAD only involves the calculation of dissimilarity measure based on stored parameters Λ and P calculated in the training phase thus the computational complexity is represented by O (N). On the other hand, DWT + SOM and DWT + SVM computational complexity are considered encoding the data observation using DWT at each node while the detection using SOM and OCSVM is done at the base station. Therefore, the complexity of applying DWT at each node is represented by O(e). Considering applying anomaly detection for SOM and OCSVM in an online manner will generate O(l) and $O\left(n^{3}\right)$ respectively. Therefore, the total computational complexity is O(e+l) and $O\left(m+n^{3}\right)$ for DWT + SOM and DWT + CESVM respectively.

The proposed CESVM-DR is based on the CESVM baseline scheme which evolved on the calculation of kernel gram matrix. The linear optimization problem formulation in the proposed CESVM-DR scheme makes it more feasible for WSNs application [38]. Furthermore, due to the reduced dimensions proposed in the CESVM-DR schemes the computational complexity of the CESVM is minimized. Although EOOD is based on the hyper-ellipsoidal SVM, it still involved the covariance matrix calculation same as the proposed CESVM-DR. The idea of EOOD is proposed to model the hyper-ellipsoid SVM in the input space, instead of the model in the feature space to reduce the complexity. Meanwhile, PCCAD and kPCA schemes are derived from the PCA algorithm. Both schemes are tuned to suit one class PCA while PCCAD involves dimension reduction and kPCA is based on kernel computational. Meanwhile, Discrete Wavelet Transform (DWT) is integrated into both DWT + SOM and DWT + OCSVM schemes as the data compression approach. In DWT + SOM and DWT + OCSVM schemes, the whole compressed data is sent to the base station for detection. Thus, both DWT + SOM and DWT + OCSVM are also utilizing the reduction of data size technique without losing significant features of the data. However, the limitation of both schemes is adopted batch learning that can affect the computational complexity.

#### 5.2.3. Communication Overhead

Communication overhead represents the amount of data communication with the network either between sensor nodes or communication between the sensor nodes and the cluster head or base station. As the anomaly detection is done in the local node, no communication overhead is incurred for the proposed CESVM-DR. The same goes for PCCAD and EOOD where detection is done at the sensor nodes. Meanwhile, the baseline CESVM scheme is based on a centralized anomaly detection where the whole set of data measurements is communicated to the base station. This is represented as O(np) when communication is done between a pair of sensor nodes. The CH receives data vectors periodically from sensors in kPCA detection model, thus communication overhead to transmit the whole data is represented as O(np). For DWT + SOM and DWT + CESVM, communication of wavelet coefficient to the central node is represented as O(mk) where k represents the wavelet coefficient.

## 6. Conclusions

Designing an effective anomaly detection scheme for WSNs is crucial yet challenging due to the limited resources. The aim is to prolong the sensor connectivity by reducing energy consumption and improving memory utilization while maintaining accurate data at the base station. Due to the ability of unsupervised classification technique to classify unlabeled data as normal or anomalous, the One-Class Support Vector Machine (OCSVM) technique is used as the classifier to design the anomaly detection scheme. The OCSVM variants namely the Centered hyper-Ellipsoidal Support Vector Machine (CESVM) has been formulated based on a linear programming approach while considering multivariate data is used as OSVM kernel to reduce computation costs. However, the parameter tuning to perform the anomaly detection must be tuned properly to suit the datasets for better performance results especially regularization parameter in dynamic data. The proposed lightweight anomaly detection scheme is more efficient and effective compared with the baseline scheme centered hyper-ellipsoidal Support Vector Machine. The complexity of the baseline centered hyper-ellipsoidal Support Vector Machine is further reduced by incorporating of the Candid Covariance-Free Incremental Principal Component Analysis (CCIPCA) algorithm to minimize the computation complexity of the CESVM algorithm. Thus, by applying CCIPCA algorithm the high communication cost due to the broadcasting of the entire covariance matrix among the nodes of the network is reduced in the proposed lightweight anomaly detection scheme. Results of the experiments show higher consistency in detection accuracy and detection rate in all of the experiments either using multivariate or univariate datasets. The comparable false alarm rate is reported throughout the evaluation which was performed with the other anomaly detection schemes. In terms of detection efficiency, with the incorporation of CCIPCA dimension reduction techniques, the communication complexity is reduced compared to the original CESVM anomaly detection scheme.

One of the limitations of the proposed lightweight anomaly detection scheme is the ability to globally assure the real anomalous nature of normal events during the anomaly detection process. The effectiveness and efficiency of the proposed anomaly detection scheme can be further improved by designing the distributed anomaly detection using spatial correlation of sensor nodes in the cluster to construct a global normal reference. Meanwhile, dynamic changes in deployed environments have an impact on the effectiveness of anomaly detection models. As the reference normal model becomes rigid over time, adaptive learning of the model is required to ensure the quality of sensor measurements.

## Figures and Tables

**Table 1:** Statistical characteristics for normal and generated artificial anomalies for GSB datasets.

Dataset	Variable	Normal		Anomalies	
		Mean	Std. Dev	Mean	Std. Dev
D1	Ambient temperature Relative humidity	5.26 25.43	8.28 0.99	7.75 33.83	9.90 0.86
D2	Ambient temperature Relative humidity	3.61 14.24	7.44 1.97	5.39 20.88	9.00 4.11
D3	Ambient temperature Relative humidity	3.29 20.27	6.86 0.74	5.33 27.52	9.77 1.00
D4	Ambient temperature Relative humidity	4.56 10.22	8.15 2.94	7.69 15.14	10.02 5.09
D5	Ambient temperature Relative humidity	3.37 24.71	7.92 1.29	10.65 33.08	11.60 1.34

**Table 2:** Performance comparison between the proposed CESVM-DR scheme and related anomaly detection schemes using simulated labelling with a kernel width of 2.

Measure	Scheme	D1	D2	D3	D4	D5	Average
DR (%)	CESVM-DR	96.4	92	92	100	100	96.08
	CESVM	74.8	85.2	82	78.8	80.8	80.32
	EOOD (local)	100	100	100	100	100	100
	kPCA(local)	100	100	100	100	100	100
FPR (%)	CESVM-DR	1.2	1.2	1.2	1.2	1.2	**1.2**
	CESVM	1.2	1.2	1.2	1.2	1.2	1.2
	EOOD (local)	32.7	32.7	40.9	34.5	30.5	34.26
	kPCA(local)	7.4	37.6	47.2	35.4	7.4	27
FNR (%)	CESVM-DR	3.6	8	8	0	0	**3.92**
	CESVM	25.2	14.8	18	21.2	19.2	19.68
	EOOD (local)	0	0	0	0	0	0
	kPCA(local)	0	0	0	0	0	0
Accuracy (%)	CESVM-DR	98.6	98.2	98.2	98.9	98.9	**98.56**
	CESVM	96.6	97.6	97.3	97	97.2	97.14
	EOOD (local)	70.3	70.3	62.8	68.6	72.3	68.86
	kPCA(local)	93.3	65.3	57.1	64	63.6	68.66

**Table 3:** The Comparison Proposed of Effectiveness Evaluation with Other Related Anomaly Detection Schemes Using Histogram-Based Labelling.

Dataset	Model	DR	ACC	FPR	FNR
IBRL	DWT + OCSVM	100	98.3	1.9	0
	DWT + SOM	100	99	1.09	0
	PCCAD	100	99.7	0.3	0
	CESVM-DR	100	98.4	1.6	0
LUCE	DWT + OCSVM	100	98.3	1.9	0
	DWT + SOM	100	99	1.09	0
	PCCAD	100	99.9	0.09	0
	CESVM-DR	100	98	2	0
PDG	DWT + OCSVM	99.7	97.6	2.6	0.3
	DWT + SOM	83	97.8	0.5	16.5
	PCCAD	97.9	96.7	3.5	2.1
	CESVM-DR	99.1	78.6	25.8	0.01
NAMOS	DWT + OCSVM	100	88.6	12.8	0
	DWT + SOM	100	99.4	0.5	0
	PCCAD	100	90.2	11.5	0
	CESVM-DR	100	100	0	0

**Table 4:** The efficiency evaluation between CESVM-DR and other schemes.

Scheme	Memory Utilization	Computational Complexity	Communication Overhead
CESVM	O (mn+np)	$O\left(n^{2}+m^{2}n\right)$	O(np)
EOOD	O(np)	$O\left(P+mp^{2}\right)$	-
PCCAD	O(nd)	O(Nd)	-
kPCA	O(k+np)	$O\left(np^{2}\right)$	O(np)
DWT + SOM	O (n)	O(e+l) (online)	O(mk)
DWT + OCSVM	O (n)	$O\left(e+s^{3}\right)\;\left(online\right)$	O(mk)
CESVM-DR	O (mn+nd)	$O\;\left(P+m^{2}d+dn^{2}\right)$	-

**Table 5:** The description of efficiency parameter.

Legends	Descriptions
*m*	Low-rank approximation of the kernel Gram matrix
*n*	Number of the data observations
*p*	The dimension of the data vector
*d*	The reduced dimension of the data vector
*P*	linear optimization problem calculation
*N*	The calculation of CCIPCA
*e*	applying anomaly detection for DWT
*s*	applying anomaly detection online for OCSVM
*l*	applying anomaly detection for SOM
*k*	communication of wavelet coefficient to the central node

## Data Availability

For more information on datasets used in the experiments, please visit: GSB, Grand-St-Bernard (GSB) dataset, 2007. http://lcav.epfl.ch/cms/lang/en/pid/86035, Accessed date (20 April 2018) IBRL, Intel Berkeley Research Lab Dataset, 2004. http://db.csail.mit.edu/labdata/ labdata.html, Accessed date (26 September 2017) PDG, Patrouille des Glaciers dataset, 2008. http://lcav.epfl.ch/cms/lang/en/pid/86035, Accessed date (23 April 2016) LUCE, Lausanne Urban Canopy Experiment, 2007. http://lcav.epfl.ch/cms/lang/en/pid/86035, Accessed date (24 January 2018) NAMOS, Networked Aquatic Microbial Observing System Dataset, 2006. http://robotics.usc.edu/~namos/data/, Accessed date (12 October 2017).
